# Odours Influence Visually Induced Emotion: Behavior and Neuroimaging

**DOI:** 10.3390/s100908185

**Published:** 2010-09-01

**Authors:** Peter Walla, Lüder Deecke

**Affiliations:** 1 School of Psychology, Newcastle University, Callaghan 2308 NSW, Newcastle, Australia; 2 Clinic of Neurology, Medical University Vienna, Währinger Gürtel 18-20, 1090 Vienna, Austria; E-Mail: lueder.deecke@meduniwien.ac.at (L.D.); 3 Ludwig Boltzmann Institute for Functional Brain Topography, Vienna, Austria; 4 Department of Psychology, Biological Psychology, University of Vienna, Liebiggasse 5, 1010 Vienna, Austria; 5 Neuroconsult e.U., Applied Neuroscience Institute, Güntergasse 3/3, 1090 Vienna, Austria

**Keywords:** emotion, olfaction, interaction, behavior, brain imaging

## Abstract

The present study was conducted to investigate the influence of olfaction on subjective valence intensity ratings of visual presentations. Pictures of five different categories (*baby, flower, erotic, fear and disgust*) were presented each being associated with five different odour conditions [no odour, low and high concentrations of phenylethyl alcohol (positive odour) and low and high concentrations of hydrogen sulphide (negative odour)]. Study participants had to rate the emotional content of each picture with respect to valence and intensity while brain activities were recorded with a whole-cortex magnetoencephalograph (MEG). A significant interaction between odour condition and picture category with respect to rating performance was found. In particular, positive valence intensity ratings related to *flowers* were increased in positive and negative odour conditions. Negative valence intensity ratings related to *disgusting* pictures were also increased in positive and negative odour conditions. The only decrease was found in the *baby* category in the high concentration negative odour condition. No behavioural effects were found for the categories *erotic* and *fear*. Around 300 ms after stimulus onset odour-related brain activity effects were found for all picture categories. On the other hand, around 700 ms after stimulus onset odour-related brain activity effects occurred only in the *flower*, *fear* and *disgust* picture categories. We interpret that early information processing demonstrates more pronounced olfactory and visually induced emotion interaction than later information processing. Since the early time window more likely reflects subconscious information processing we interpret that interaction between olfaction and visually induced emotion mostly occurs below the level of consciousness. Later, rather conscious information processing, seems to be differently influenced by simultaneous olfaction depending on the kind of emotion elicited through the sense of vision.

## 1. Introduction

Emotion is a scientific field which has received a lot of attention and yet as it seems its overall picture is not the clearest to date. At the same time, olfaction has long been neglected in human brain research but now seems to become a more important topic, perhaps due to its obvious link to emotion. In particular, two aspects related to the sense of smell are exciting in relation to emotion. First, it has a strong subliminal component [1–4] and second, it is heavily connected with emotional memory [5,6]. For example, it was demonstrated that memories recalled by odours were more emotional and evocative than those recalled by the same cue but presented visually or acoustically [5]. Also, the idea was supported that olfactory hedonic responses are learned through emotional associations [7]. This means that odour hedonic perception and odour-related behaviour result from a learned association between an odour and the emotional content in which the odour was first encountered. As a matter of fact, there is quite some neuroanatomical overlap regarding olfaction and emotion. In the end, it must mean something that odour-related information enters the limbic system via the amygdale after just two synapses while at the same time the amygdale process negative emotion and sit at the gate to the hippocampus which in turn is heavily involved in long term memory. Previous physiological investigations revealed that bilateral amygdala activation is elicited by odours, regardless of valence [8]. In the posterior orbitofrontal cortex, neural responses evoked by pleasant and unpleasant odours were segregated within medial and lateral segments, respectively. The same authors further suggested that this indicates functional heterogeneity in areas critical to human olfaction. They also show that brain regions mediating emotional processing are differentially activated by odour valence, providing evidence for a close anatomical coupling between olfactory and emotional processes. An fMRI study provides evidence that the effect of odour-related intensity on amygdale activity is not the same at all levels of valence [9]. In particular, the amygdale responds differentially to high- *versus* low-intensity odour for pleasant and unpleasant odours but not for neutral odours. The authors inferred that the amygdale codes neither intensity nor valence per se, but a combination which reflects the overall emotional value of a stimulus.

In spite of various solid reports, it remains unclear how close emotion and olfaction really are, but there is accumulating evidence about interactions between the two. For example, the influence of affective pictures on olfactory functions was investigated [10]. The researchers found that after viewing positive pictures an odour was rated significantly more pleasant while an odour was rated less pleasant and more intense after the presentation of unpleasant pictures. The authors concluded that inducing a negative emotional state by visual stimulation reduces olfactory sensitivity. In those studies, visually induced emotion was the controlled independent variable and odour rating was the measure. On the other hand, it also seems important to investigate influences of olfaction on visually induced emotion. In a systematic review, credible evidence that odours can affect mood, physiology and behaviour has been reported [11], but how do odours influence the rating of distinct visually induced emotion? It was further proposed that the temporal pole binds perceptual inputs to visceral emotional responses and because perceptual inputs remain segregated into dorsal (auditory), medial (olfactory) and ventral (visual) streams, the integration of emotion with perception is channel specific [12]. This suggests that emotional aspects of odours influence emotional aspects of pictures. Zhou and Chen reported about the integration of emotional cues from vision and olfaction [13]. They suggested that specifically fear-related chemical signals modulate human’s visual emotion perception in an emotion-specific way. Behavioral studies about ratings related to facial attractiveness demonstrated that simultaneous olfaction has an influence on subjective preference [14]. After all, an investigation about olfactory influences on emotionally loaded pictures of various categories by using brain imaging technology seems promising.

Our study aimed at a basic investigation about olfactory influences on visually induced emotion while measuring brain activities. We were interested in providing accurately controlled olfactory stimulation with a computer controlled olfactometer by using low and high concentrated positive and negative odours compared to an odourless control condition. During odour stimulation with high temporal resolution emotionally loaded pictures from five different categories were presented. Subjective valence intensity ratings under all five different olfactory conditions were used as dependent variables. Our motivation simply was to demonstrate that the same olfactory conditions have different effects on simultaneous visually induced emotion depending on the emotional content. We recorded brain activity with a whole-cortex magnetoencephalograph (MEG) to describe neurophysiological processes related to interactions between visually induced emotion and olfactory stimulation. Due to previous findings about chronological brain activities related to subconscious and conscious olfactory information processing different odour-related effects on visually induced emotion depending on time after stimulus onset were expected.

## 2. Methods

### 2.1. Participants

Ten volunteers (five females) were paid for participation. Their mean age was 24.3 (SD = 2.9) and all were right handed and without any neuropathological history. They all had normal or corrected to normal vision (contact lenses) and gave their written informed consent.

### 2.2. Procedure

**Figure 1.**
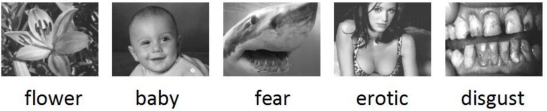
Example pictures for each category.

The subjects were seated on a comfortable chair and viewed a screen in front of them onto which a preselected collection of pictures was presented (distance from eyes to screen was around 1.70 m and the visual angle was about 3.5° vertical and 5° horizontal). Each picture appeared centrally for 1,000 ms with an inter stimulus interval of 4.6 s. Between each picture a random noise picture was shown to minimize contrast differences between picture stimulation and inter stimulus time. Shortly before each picture a plus (+) was shown as a fixation point (overlaid on the noise picture). In total, 750 grey scale pictures were shown during one experiment which consisted of six sessions, each session containing 125 picture presentations (5 picture categories ×5 olfactory conditions ×5 pictures per category). Pictures were selected according to the categories baby, flower, disgust, erotic and fear (Figure 1) assuming to cover a certain variety of valence and arousal. Pre-evaluation related to valence and arousal was not necessary because we were interested in relative within-subject effects.

All participants were given the instruction to rate every single picture presentation on a scale from minus 5 to plus 5. The minus to plus dimension reflected valence direction whereas 1 to 5 was used for intensity ratings within either valence direction. They were all informed about olfactory stimulation. However, it was emphasized that any olfactory stimulation is not part of the task and no attention needs to be paid to odor stimuli.

Each picture was simultaneously associated with one of the five olfactory conditions in random order. To enable the delivery of 1s olfactory stimuli we used a computer controlled olfactometer.

### 2.3. Olfactory stimulation

An olfactometer which was produced by Burghart Messtechnik GmbH (Germany) was used for nasal chemical stimulation. It embeds pulses of an odourant into a constantly flowing air stream of a consistent temperature and relative humidity (36.5 °C, 80% relative humidity). Its stimulus characteristics can be optimized so that the rise time does not exceed 20 ms. To ensure a constant odour delivery in the right nostrils of all study participants they were trained to breathe through the mouth to avoid respiratory airflow in the nose. In case of chemical stimulation a constantly flowing airstream (7.3 L/min; control air) including neutral room air was replaced by an airstream containing one of the chemical stimuli for 1,000 ms. The control condition was to replace the constant control airstream by the stimulus airstream but without containing a chemical stimulus.

To mask the slight acoustic signal during the switch between control air and stimulus air acoustic white noise of about 80 dB SPL was applied to both ears of each participant. Olfactory stimuli were two concentrations of phenylethyl alcohol (PEA; 100% PEA and 5% PEA + 95% control air) known as rose flavor and two concentrations of hydrogen sulfide (H_2_S) (about 3 ppm and about 0.03 ppm) known as smell of rotten eggs.

### 2.4. Recordings

The MEG was a 143 channel whole-cortex-system manufactured by CTF Systems Inc. (Canada). The sampling rate was 250/s. Recordings were filtered online with a band pass from 0.3 Hz to 80 Hz. An offline band pass filter from 0.5 Hz–30 Hz was applied (offline digital filters were zero phase filters; CTF Systems Inc.). Average event-related fields (ERFs) were calculated for each participant and across all participants for each encoding condition. Intra individual head positions were not changed between experimental sessions and inter individual differences were kept as small as possible. ERF curves overlaid across all 143 sensors are used to visualize neurophysiological brain activity patterns of all experimental conditions.

### 2.5. Statistics

For statistical analysis of physiological data the mean amplitudes of 100 ms intervals (overlapping for 50 ms) covering the time range from stimulus onset (0 ms) to 1,000 ms after stimulus onset were determined for each participant, each condition and each sensor. All resulting data were also normalized. Raw data are interpreted as to reflect quantitative physiology whereas normalised data are interpreted as to reflect qualitative physiology. A 3-way repeated measures design ANOVA (within subjects) was then applied to all raw data and to all normalized data for each separate emotion category and for every single time interval. Thereby, the factors *odour, hemi* and *sensor* were introduced. The consecutive ANOVA results (from 0 ms to 1,000 ms after stimulus onset) were then used to demonstrate changes in the significance of odour-related effects over time. Furthermore, for each emotion category the control condition was separately compared to every single olfactory condition (ANOVAs). This was done to investigate neurophysiological differences between separate olfactory conditions in more detail. No clear pattern was found. We therefore decided to exclude any detailed report about respective results. However, it helped us to define the condition with maximum effects. Behavioral data were also calculated by means of ANOVA.

## 3. Results

### 3.1. Behavioural results

**Figure 2.**
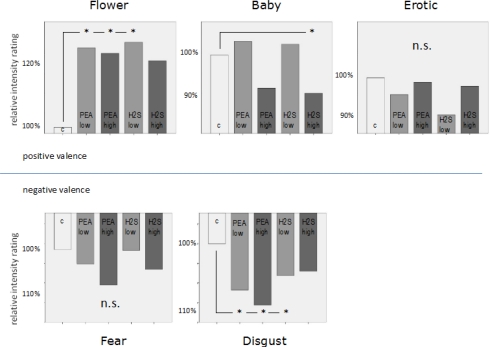
Bar diagrams for each picture category demonstrating differences in valence intensity rating for all olfactory conditions in relation to olfactory control.

A 2-way ANOVA including *emotion* and *odour* as factors revealed a highly significant *emotion* main effect (F = 85.606; p < 0.001; Greenhouse-Geisser corrected). This result confirms that the picture categories which we used in the present study did indeed elicit different emotional perceptions. In addition, a significant *emotion x odour* interaction (F = 3.283; p = 0.013; Greenhouse-Geisser corrected) related to the dependent variables of valence intensity rating shows that these emotional perceptions were also manipulated by the different olfactory conditions provided in this study. See Figure 2 showing bar diagrams of all rating performances.

Further analysis revealed a significant subjective emotion rating decrease for the emotional category *baby* in case of *H_2_S high concentration* (T = 2.306, p = .047) compared to the neutral condition. For the emotional category *flower* we found significant increases in subjective emotional rating for the chemical conditions *PEA low concentration* (T = −2.826, p = 0.020), *PEA high concentration* (T = −2.742, p = 0.023) and *H_2_S low concentration* (T = −2.913, p = 0.017) compared to the neutral condition. *H_2_S high concentration* showed a certain trend in the same direction of an increase in subjective emotional rating but without statistical significance (T = −1.788, p = 0.107) compared to the neutral condition. For the emotional category *erotic* we did not find any significant differences related to our different chemical stimulation conditions. However, qualitatively there is a trend towards subjective emotional rating reductions for all chemical conditions compared to the neutral condition. For *fear* as an emotional category statistical analysis also revealed no significant differences for all separate comparisons between every single chemical stimulation condition with the neutral condition. Only a slight trend was found for the chemical condition *PEA high concentration*. Finally, for the emotional category *disgust* we found significant increases in subjective negative emotional rating associated with chemical conditions of *PEA low concentration* (T = 2.944, p = 0.016), *PEA high concentration* (T = 5.126, p = 0.001) and *H_2_S low concentration* (T = 2.574, p = 0.030) compared to the neutral condition. A very strong trend in the same direction was also found for the H_2_S high concentration chemical condition compared to the neutral condition (T = 2.034, p = 0.073). Please, find all behavioral results including percentage values related to subjective emotional rating changes in Figure 2. See also Table 1 demonstrating mean ratings plus distinct percentage numbers of rating changes due to olfactory conditions.

### 3.2. Physiological results

Statistical analysis revealed significant *odour x hemisphere x sensor* interactions for all five picture categories. A closer look at the temporal characteristics of these interactions reveals that they consistently occurred in every picture category between 250 ms and 400 ms, whereas other time frames show differences between picture categories (see Table 2a) in the raw data set. The raw data set reflects differences with respect to brain activity magnitude. Roughly, it can be assumed that significant differences in raw data demonstrate that the same neural generators were activated to a different degree (different amplitude). On the other hand, normalized data reflect functional features of brain activity. Significant differences after data normalization demonstrate that different neural generators were involved in the different conditions included in the statistics. Table 2b presents statistical results of the normalized data sets and shows that picture category erotic did not result in any significant interaction. This is interpreted as to show that no functional differences between olfactory conditions occurred in this picture category.

**Figure 3.**
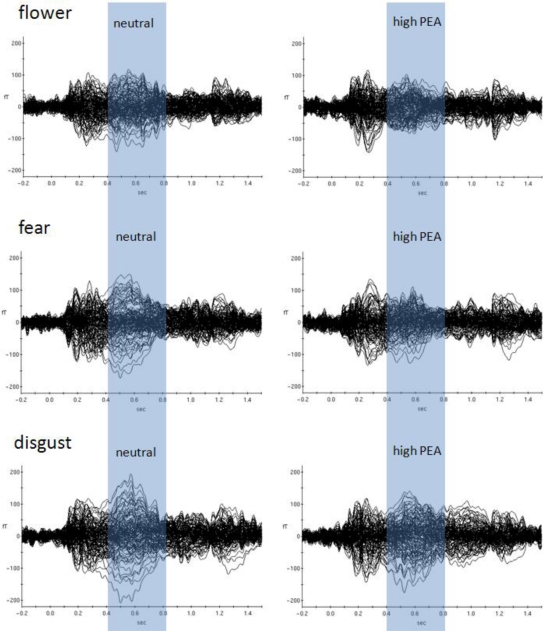
Comparison of MEG curves reflecting olfactory control during picture processing *versus* high concentration positive odour during picture processing for three picture categories which were associated with significant differences. Note that later brain activities between about 400 ms to 800 ms after stimulus onset are obviously different between the two olfactory conditions in each of these picture categories.

Later odour-related effects occurred for the picture categories *flower*, *fear* and *disgust* (Table 2 and Figure 3). Obviously, the most pronounced interactions occurred between 150 ms and 850 ms in the *flower* picture category (raw data and normalized data). According to the behavioral results, this category is associated with the most neutral valence ratings with respect to absolute values. It is therefore concluded that odour-related brain activity is most effective in situations with less visually induced emotion, at least in case of positive visually induced emotion. Clearly, both strongly positive picture categories (*erotic* and *baby*) show the shortest temporal windows of odour-related effects on brain activity. The two picture categories associated with strong negative valence ratings (*fear* and *disgust*) show evidence of later odour-related brain activity effects as well, but not as pronounced as with *flowers*. Figure 3 demonstrates dominant later odour-related effects on brain activities for the categories *flower*, *fear* and *disgust*. While the earlier effects cannot really be seen by visual inspection of ERFs the later effects clearly show reduced amplitudes in case of simultaneous olfactory stimulation in these picture categories.

## 4. Discussion

This study reports about physiological and behavioral measures of simultaneous olfactory and visual information processing. In particular, emotionally loaded pictures had to be rated with respect to valence and intensity under different olfactory conditions while brain activity was recorded with magnetoencephalography (MEG). The present findings support the idea that different visually induced emotions are differently influenced by simultaneous olfactory stimulation. First, this finding underlines that different emotions are associated with different neural substrates which differently overlap with olfactory neural pathways. In terms of functional interpretations it can be inferred that odour-induced emotions differently interfere with different visually induced emotions. Without providing further evidence we would nevertheless like to mention that we believe olfaction to be the earliest emotion system per se. According to this working hypothesis, this early emotion system developed via the sense of olfaction and was then used by other sensory modalities to evaluate their respective input. Of course further investigations are needed to test this hypothesis. Reports like, odours change the pitch of voice in humans might also point into a very basic direction of olfactory influences [15]. Recently, the idea that odour-related emotions are different compared to other known emotions was mentioned [16]. They stated that the examination of the nature of verbal labels that describe emotional effects elicited by odours point to a structure of affective responses to odours that differs from the classical taxonomies of emotion such as posited by discrete bidimensional emotion theories. The authors suggest that the subjective affective experience or feelings induced by odours are structured around a small group of dimensions that reflect the role of olfaction in well-being, social interaction, danger prevention, arousal or relaxation sensations, and conscious recollection of emotional memories. Our study provides behavioural and physiological evidence about different emotional qualities between olfaction and vision.

### 4.1. Behavioural data

Behavioural data analysis revealed various significant effects related to changes in subjective ratings of valence intensity due to simultaneous olfactory stimulation. By far the most dominant behavioural influence of simultaneous olfactory stimulation occurred in the *flower* condition. This condition happened to be the most neutral condition in terms of visually induced emotion (see Table 1 and Figure 2). The mean intensity rating for *flowers* in the olfactory control condition was 1.53 (positive) whereas it was 2.48 (positive) for *erotic* pictures and 2.59 (positive) for *baby* pictures. In the *erotic* condition no significant effect occurred. In the *baby* condition a slight significant reduction in emotion intensity rating occurred (92.7% of neutral) in case of high concentration negative odour. In the *flower* condition we found an increase in emotion intensity rating of 126.1% in case of low concentration positive odour, 124.2% in case of high concentration positive odour and 127.5% in case of low concentration negative odour. Thus, positive valence ratings in low intensity ranges seemed to be mostly affected by simultaneous olfactory stimulation.

In contrast, for visually induced negative emotion the most dominant effect occurred in the condition which was associated with highest intensity ratings, in this case *disgust*. The mean valence intensity rating for the neutral odour condition for *disgusting* pictures was 3.6 (negative). Small but significant increases of 106.7% in negative valence intensity rating occurred in the low concentration positive odour condition, 108.6% in the high concentration positive odour condition and 104.4% in the low concentration negative odour condition. No significant effects at all were found for the *fear* category which was associated with a mean intensity rating in the neutral odour condition of 2.13 (negative). Therefore, negative valence ratings in high intensity ranges seemed to be mostly affected by simultaneous olfactory stimulation.

This different pattern of behaviour-related results for positive and negative visually induced emotion underlines the notion of qualitatively different systems related to these opposite valence directions. In the positive valence direction less visually induced emotion intensity was most obviously influenced by simultaneous olfaction whereas in the negative valence direction it was high visually induced emotion intensity. Interestingly, in a previous fMRI study it was found that emotion-related arousal and pleasantness related to odors are processed independently by different brain regions [17]. More importantly the authors demonstrated that for olfaction, the amygdale had no intrinsic preference for negative emotion after the effects of arousal had been controlled. This could explain why in our study olfactory influences occurred at particular intensity levels which were different between positive and negative visually induced emotions. A further interpretation of our behavioural results is that the negative emotion of fear was not affected by simultaneous olfactory stimulation in terms of modifying intensity ratings because it heavily involved amygdale activation. As already mentioned in the introduction, the amygdale are also known for their involvement in olfactory information processing, especially at early processing stages. We can therefore infer that both sensory modalities engaged the amygdale and subjective rating of visually induced emotion occupies these neural structures more dominantly than olfaction not allowing it to interfere.

### 4.2. Physiological data

Physiologically, we investigated whether the different olfactory conditions elicited different patterns of effects with respect to our five picture emotion categories. After all, the analysis of brain activity data revealed different patterns of significant effects across emotion categories demonstrating different influences of simultaneous olfactory stimulation on brain processes engaged in different emotion-related information processing triggered through the sense of vision. The fact that we introduced hemisphere as a factor in our statistical analysis goes back to previous findings about lateralisation related to odour information processing. Previous work about hemispheric differences related to odour processing in the human brain has been summarised [18]. The authors found that the left hemisphere participates more in processing emotion-related information of an olfactory stimulus than the right hemisphere. On the other hand, differential hemisphere involvement with a right-side advantage for processing on unpleasant affect in olfaction was suggested [19]. These inconsistent findings left us with the supportive idea to include hemisphere in our statistical analysis but we did not further analyse our data with respect to lateralisation phenomena. Thus, we only report about the principal physiological finding that odours have different effects on different visually induced emotions.

Although in our study differences occurred between emotion categories there is evidence for at least one common effect in all of them at an early processing stage. Repeated measures ANOVAs including all five olfactory conditions (control, low concentration PEA, high concentration PEA, low concentration H_2_S and high concentration H_2_S) and separately calculated for each visual emotion category and for each individual 100 ms time window revealed common significances across emotional categories between about 250 ms and 400 ms after stimulus onset. Only in the normalized data set in the emotion category *erotic* no such significances occurred. Anyway, this early time window is known to reflect brain activities engaged in subconscious olfactory information processing [2–4]. It can therefore be concluded that subconscious olfaction-related information processing most consistently varies across all five picture emotion categories. However, as mentioned above, the only emotion category without significant olfaction-related effects in this early time window in the normalized data set was *erotic*. It has to be emphasized though that respective p-values show a strong trend towards significance, but they were above the threshold of 0.05. This is why this emotion category deserves further consideration, especially, because behavioural data analysis also revealed no significant effect in the *erotic* emotion category. This coincidence points to a correlation between brain activities and behaviour, however, we cannot draw this general conclusion, because the rest of the categories does not show any sign of such a correlation. Nevertheless, the fact that neither physiological recordings related to erotic images nor subjective valence intensity ratings related to them were influenced by simultaneous olfactory stimulation is puzzling. One possible explanation for this finding is that the direct relation of erotic content to conservation of the own species is so robust that its elicited emotion is not that vulnerable to simultaneous olfaction as in other picture emotion categories. This notion is underlined by the fact that subjective valence intensity ratings related to the emotion category of *fear* weren’t affected by simultaneous olfaction either. Fear is directly related to the conservation of oneself which might also reflect a rather robust process not easily influenced by emotion elicited through other sensory modalities. However, physiologically the emotion category *fear* was associated with significant olfactory effects, in contrast to the emotion category *erotic*.

Most obviously, different patterns of significant olfaction-related differences between picture emotion categories occurred during later time intervals (Figure 3). Whereas the early time window around 300 ms after stimulus onset reflects subconscious olfactory information processing, later intervals around 700 ms after stimulus onset were previously found to reflect conscious odour perception [2–4]. We found three emotion categories to be associated with significant olfaction-related influences in this later time window, *flower*, *fear* and *disgust*. Although varying a bit in terms of exact temporal occurrence only these emotion categories showed significant olfaction-related effects after the earlier consistent effect which was found for all emotion categories. This finding provides evidence for different conscious odour-related influences on these emotion categories only. Figure 3 shows event-related magnetic field (ERF) distributions for the neutral olfactory condition and the high concentration PEA olfactory condition (most dominant physiological effects were found for PEA high concentration) of these three emotion categories. As can be seen, in all three emotion categories high concentration PEA stimulation resulted in reduced ERF amplitudes in later time intervals. This physiological situation corresponds with increases in intensity ratings irrespective of valence direction. However, in case of the emotion category *fear* increases in intensity rating were not significant.

The emotion category *flower* exhibited most pronounced later olfactory-related brain activities and it was also the *flower* category which was most dominantly affected with respect to changes in valence intensity ratings. We can therefore infer that the later component reflecting conscious odour perception was mostly responsible for the significant behavioural changes. Only simultaneous conscious odour perception modifies explicit subjective ratings related to visually induced emotion. A similar conclusion was previously drawn [3] in a study about olfaction and word processing. In that study, it was concluded that only simultaneous conscious olfaction during deep word processing caused reductions in later word recognition performance. Further evidence for olfaction and language interactions was also presented earlier [20].

As already mentioned, separate comparisons between the chemical control condition and every single olfactory condition for each emotion category revealed the most pronounced effects for the high concentration PEA (phenylethyl alcohol) condition. At the moment we don't want to further discuss detailed effects of particular odour conditions, but it seems that further investigations focusing on this issue might lead to promising results with respect to a better understanding of emotions related to olfaction.

After all, the present study provides evidence of variable interference between olfaction and visually induced emotion depending on what emotion is visually processed and depending on its perceived intensity. Follow up studies are planned with more study participants to calculate possible gender differences. It is well known that gender plays an important role in olfaction and maybe some of the data variability leading to insignificant results goes back to gender differences. Also, better control of valence and intensity (arousal) related to picture presentations will help to better understand selective influences related to both dimensions.

## Figures and Tables

**Table 1.** Mean valence intensity ratings related to all picture categories and for every single olfactory condition. In addition, relative changes in rating performance are displayed in %.

	**Baby**	**Flower**	**Erotic**	**Fear**	**Disgust**
**Control**	2.59; 100%	1.53; 100%	2.48; 100%	−2.13; 100%	−3.60; 100%
**low PEA**	2.66; 102.7%	**1.93; 126.1%**	2.39; 96.4%	−2.20; 103.3%	**−3.84; 106.7%**
**high PEA**	2.43; 93.8%	**1.9; 124.2%**	2.45; 98.8%	−2.31; 108.5%	**−3.91; 108.6%**
**low H_2_S**	2.64; 101.9%	**1.95; 127.5%**	2.29; 92.3%	−2.13; 100%	**−3.76; 104.4%**
**high H_2_S**	**2.40; 92.7%**	1.86; 121.6%	2.44; 98.4%	−2.23; 104.7%	−3.74; 103.9%

**Table 2.** Reapted measures ANOVA results of raw and normalised MEG data. Displayed are p-values after Greenhouse-Geisser correction for odor*hemisphere*sensor interaction with each picture category and for all time intervals. Note that between 250 ms and 400 ms after stimulus onset consistent effects occurred across picture categories (marked in dark grey). Other significant interactions are marked in light grey.

**(a)** Raw data.																	
ms	0– 100	50– 150	100– 200	150– 250	200– 300	250– 350	300– 400	350– 450	400– 500	450– 550	500– 600	550– 650	600– 700	650– 750	700– 800	750– 850	800– 900
FLOWER	0.174	0.222	0.188	**0.043**	**0.007**	**0.004**	**0.013**	**0.041**	**0.043**	**0.036**	**0.038**	**0.044**	**0.030**	**0.030**	**0.043**	**0.048**	0.067
EROTIC	0.619	0.484	0.360	0.345	0.181	**0.035**	**0.020**	**0.045**	0.107	0.074	0.180	0.225	0.311	0.277	0.391	0.541	0.415
BABY	0.215	0.145	0.387	0.378	0.069	**0.007**	**0.006**	**0.013**	0.142	0.**049**	0.146	0.208	0.224	0.056	0.082	0.167	0.175
FEAR	0.232	0.211	0.247	0.307	**0.019**	**0.003**	**0.021**	**0.049**	**0.034**	0.339	**0.017**	**0.026**	**0.041**	0.344	0.368	0.346	0.212
DISGUST	0.429	0.438	0.445	0.343	**0.006**	**0.002**	**0.020**	0.106	0.062	**0.028**	**0.048**	0.076	0.185	0.056	**0.044**	0.097	0.344

**(b)** Normalised data.																	
ms	0– 100	50– 150	100– 200	150– 250	200– 300	250– 350	300– 400	350– 450	400– 500	450– 550	500– 600	550– 650	600– 700	650– 750	700– 800	750– 850	800– 900
FLOWER	0.208	0.295	0.201	**0.035**	**0.013**	**0.015**	**0.022**	**0.036**	**0.033**	**0.022**	**0.030**	0.071	0.064	**0.040**	**0.044**	0.068	0.074
EROTIC	0.728	0.605	0.302	0.543	0.308	0.052	0.061	0.280	0.280	0.285	0.376	0.371	0.321	0.324	0.450	0.650	0.616
BABY	0.355	0.181	0.364	0.273	0.053	**0.001**	**0.003**	**0.011**	0.097	0.082	0.065	0.096	0.190	0.059	0.054	0.088	0.156
FEAR	0.315	0.222	0.405	0.371	**0.040**	**0.008**	**0.022**	**0.047**	**0.032**	**0.038**	**0.044**	**0.029**	0.052	0.136	0.200	0.280	0.350
DISGUST	0.429	0.438	0.445	0.343	**0.006**	**0.002**	**0.020**	0.106	0.062	**0.028**	**0.048**	0.076	0.185	0.056	**0.044**	0.097	0.344
